# Mining a differential sialotranscriptome of *Rhipicephalus microplus* guides antigen discovery to formulate a vaccine that reduces tick infestations

**DOI:** 10.1186/s13071-017-2136-2

**Published:** 2017-04-26

**Authors:** Sandra R. Maruyama, Gustavo R. Garcia, Felipe R. Teixeira, Lucinda G. Brandão, Jennifer M. Anderson, José M. C. Ribeiro, Jesus G. Valenzuela, Jana Horackova, Cecília J. Veríssimo, Luciana M. Katiki, Tamy M. Banin, Amanda F. Zangirolamo, Luiz G. Gardinassi, Beatriz R. Ferreira, Isabel K. F. de Miranda-Santos

**Affiliations:** 1 Departament of Biochemistry and Immunology, Ribeirão Preto School of Medicine, University of São Paulo, Ribeirão Preto, SP 14049-900 Brazil; 2 Department of Genetics and Evolution, Federal University of São Carlos, São Carlos, SP 13565-905 Brazil; 3 Laboratory of Malaria and Vector Research, National Institute of Allergy and Infectious Diseases, Bethesda, MD 20852 USA; 4 Faculty of Biological Sciences, University of South Bohemia, Ceske Budejovice, 37005 Czech Republic; 5 São Paulo Institute of Animal Science, Nova Odessa, SP 13460-000 Brazil; 6 Ribeirão Preto School of Nursing, University of São Paulo, Ribeirão Preto, SP 14049-902 Brazil; 7 LGB: Faculdade de Tecnologia de Araçatuba, Araçatuba, SP 16052045 Brazil; 8 LGG: Division of Pulmonary Allergy & Critical Care Medicine, Department of Medicine, Emory University, Atlanta, GA 30322 USA

**Keywords:** *Rhipicephalus microplus* tick, Sialotranscriptome, Anti-tick vaccine, Antigen discovery, Salivary proteins

## Abstract

**Background:**

Ticks cause massive damage to livestock and vaccines are one sustainable substitute for the acaricides currently heavily used to control infestations. To guide antigen discovery for a vaccine that targets the gamut of parasitic strategies mediated by tick saliva and enables immunological memory, we exploited a transcriptome constructed from salivary glands from all stages of *Rhipicephalus microplus* ticks feeding on genetically tick-resistant and susceptible bovines.

**Results:**

Different levels of host anti-tick immunity affected gene expression in tick salivary glands; we thus selected four proteins encoded by genes weakly expressed in ticks attempting to feed on resistant hosts or otherwise abundantly expressed in ticks fed on susceptible hosts; these sialoproteins mediate four functions of parasitism deployed by male ticks and that do not induce antibodies in naturally infected, susceptible bovines. We then evaluated in tick-susceptible heifers an alum-adjuvanted vaccine formulated with recombinant proteins. Parasite performance (i.e. weight and numbers of females finishing their parasitic cycle) and titres of antigen-specific antibodies were significantly reduced or increased, respectively, in vaccinated versus control heifers, conferring an efficacy of 73.2%; two of the antigens were strong immunogens, rich in predicted T-cell epitopes and challenge infestations boosted antibody responses against them.

**Conclusion:**

Mining sialotranscriptomes guided by the immunity of tick-resistant hosts selected important targets and infestations boosted immune memory against salivary antigens.

**Electronic supplementary material:**

The online version of this article (doi:10.1186/s13071-017-2136-2) contains supplementary material, which is available to authorized users.

## Background

Infestations with ticks cause enormous losses in livestock. *Rhipicephalus microplus*, the most important species of tick affecting cattle worldwide, is predominantly found in tropical and subtropical regions [1]; it is a monoxene tick, i.e. it spends its approximately 21-days parasitic cycle (larvae, nymphs and adults) on the same host. Thereafter, most of the engorged females detach from the host to begin laying thousands of eggs in the pasture. Current control strategies primarily involve the use of acaricides leading to acaricide-resistant ticks, environmental pollution and meat and milk contaminated with residues [2]. These concerns reduce the usefulness of this approach, and thus alternative strategies for the control of tick infestations are being evaluated. Tick bites stimulate host immune responses [3], indicating that the induction of protective immunity against ticks might be achieved using vaccines and immunobiological control of these pests is feasible. The commercially available anti-tick recombinant vaccines, TickGard [4] and GAVAC [5], are based on a tick gut glycoprotein, Bm86, and the concept of hidden antigens. Hidden antigens, to which hosts are not exposed to during natural infestations, were considered to be superior to salivary-exposed antigens based on the rationale that parasites would not have developed escape mechanisms from host immune responses against them [6, 7], in spite of the fact that tick saliva mediates most mechanisms of parasitism [8]. However, while these vaccines do reduce parasitism, their efficacy proved too variable and the memory induced by them short-lived [9–12], i.e. less than ideal for production systems for livestock. Therefore, the search for new tick antigens and new strategies for antigen selection is essential to improve the control of tick infestation through vaccination.

The discovery of protective antigens for the development of a new cattle tick vaccine depends on rational strategies, and many efforts have been undertaken [13]. The post-genomic era facilitated the rational design of safer and effective vaccines based on the genome, proteome or transcriptome of parasites [14]. Here, we focus on the analysis of a differential sialotranscriptome from ticks fed on susceptible or resistant hosts, as a strategy for antigen discovery, because tick saliva is the main mediator of parasitic haematophagy [8]. In addition, exposure of vaccinated animals to saliva during natural challenges will boost immunological memory [15], thus avoiding the problems seen with vaccines formulated with non-salivary antigens. Our hypothesis for developing a tick vaccine is that it must be formulated with a cocktail of tick salivary antigens to weaken the gamut of parasitic strategies. We also hypothesise that host immunity modulates gene expression in ticks, including important vaccine targets. We generated a *R. microplus* tick transcriptome to be our catalogue for antigen discovery in order to explore important information and, consequently, vaccine targets that are not covered by the available cattle tick databases (BmiGI [16] and CattleTickBase [17]), i.e. the sequencing data obtained with ticks feeding on tick-resistant and tick-susceptible hosts. Although CattleTickBase is a very comprehensive database for *R. microplus*, sequencing data related to ticks fed on Holstein and Nelore bovines (the most common taurine and zebuine breeds in Brazil, respectively), has not been generated yet, therefore, the sequence data produced here will significantly enlarge the repertoire of ESTs for this species of tick.

Our catalogue was generated with cDNA libraries constructed with salivary glands of ticks undergoing different conditions, such as life stages and feeding on hosts with contrasting levels of anti-tick immunity. Regarding host immunity, bovine host breeds present differences in immunity to ticks, reflecting variable genetic backgrounds; for example, zebu cattle (*Bos taurus indicus*) are resistant to tick infestations, whereas taurine cattle (*B. t. taurus*) are susceptible to tick infestations [18–20]. The novelty of our strategy is that we considered the anti-tick immunity phenotypes of hosts to drive antigen discovery. We also assumed that transcripts encoding salivary proteins important for parasitism are efficiently expressed when ticks feed on susceptible hosts, thereby providing a successful life-cycle for the ectoparasite and representing adequate antigens for targeting. Therefore, we aimed to identify the antigens affected by host immunity for the development of a multicomponent anti-tick vaccine for testing in tick-susceptible bovines and to evaluate whether vaccination can control tick infestation. To the best of our knowledge, taking into consideration the impact of host anti-tick immunity on gene expression in a tick has never been used for the identification of new antigens for the development of an anti-tick vaccine for cattle.

Among the list of antigens, three candidate genes were expressed as N-terminal truncated recombinant tagged proteins and a fourth candidate was expressed as a full-length sequence. The putative biological functions of these proteins were related to suppression of host antibody responses *via* an immunoglobulin binding-protein; inhibition of host hemostatic responses *via* a thrombin inhibitor; possibly destruction of host extracellular matrix for the formation of a feeding pool *via* a metalloprotease; attachment of the tick to its hosts *via* a glycine-rich cement protein. The immunisation of Holstein calves (a breed highly susceptible to tick infestations) with the four test antigens significantly reduced the infestation of *R. microplus* ticks in vaccinated calves, with an efficacy of 73.2%. Two of these antigens induced a recall antibody response of antigen-specific IgG in calves exposed to tick bites (infestation). The results presented herein are a proof of principle that a reverse vaccinology pipeline guided by different levels of anti-tick immunity is a powerful strategy for the identification of promising antigens that can boost host immunity during the natural infestation, and that salivary (“exposed antigens”) proteins are useful components of cattle tick vaccine.

## Methods

### Ticks

For the construction of cDNA libraries (tick transcriptomes), feeding nymphs and male and female adults were collected from naturally infested *Bos taurus taurus* cattle (Holstein breed; the susceptible host) and *B. t. indicus* (Nelore breed; the resistant host). Salivary glands (SG) were dissected from 25 females, 25 males and 40 nymphs that fed on each type of host, and the samples were briefly washed in ice-cold PBS and immediately stored in RNALater solution (Ambion, Austin, TX, USA) for 24 h at 4 °C, followed by freezing at -70 °C until further use. Unfed larvae (UFL) of *R. microplus* ticks were obtained 3 days after hatching from eggs laid by females that had fed on resistant or susceptible bovines. The UFL were frozen at -70 °C and stored until further use.

For challenges with *R. microplus* infestations in the vaccination trial, the larvae were obtained from eggs laid by engorged female ticks collected from bovines naturally infested. These females were maintained at 28 °C and 90% relative humidity until oviposition. The egg masses were weighed at the third day of oviposition and aliquots of 500 mg (equivalent to approximately 10,000 hatched larvae) were used for artificial tick infestations with unfed larvae inserted in cotton jersey chambers, 2 weeks after the third dose of the immunisation regimen. The cattle undergoing challenge infestations were followed daily during the whole parasitic cycle (21 days).

### *Rhipicephalus microplus* sialotranscriptomes

A total of eight cDNA libraries were constructed: UFLRmS (unfed larvae hatched by females fed on susceptible hosts), UFLRmR (unfed larvae hatched by females fed on resistant hosts), SGNRmS (salivary glands of nymphs fed on susceptible hosts), SGNRmR (salivary glands of nymphs fed on resistant hosts), SGMRmS (salivary glands of males fed on susceptible hosts), SGMRmR (salivary glands of males fed on resistant hosts), SGFRmS (salivary glands of females fed on susceptible hosts) and SGFRmR (salivary glands of females fed on resistant hosts). Because of collection and dissection of fed larvae is not feasible, at this stage, we analysed gene expression of a whole extract of unfed larvae hatched from eggs laid by females fed on susceptible or resistant hosts (respectively, UFLRmS and UFLRmR). ESTs from each library (excluding rRNA, mitochondrial and low-complexity sequences) were deposited in the European Nucleotide Archive (Accession numbers LT708478–LT714108). Isolation of RNA, construction of cDNA libraries, amplification of clones (PCR using recombinant phages as templates) and sequencing were performed as described elsewhere [21, 22]. For all libraries, cDNA size fractionation was performed using Chroma-Spin 400 (Clontech Laboratories, Mountain View, CA, USA) before ligation in λTriplEx2 arms. The cDNA fractions were pooled in three main sizes named small (S), medium (M) and large (M) to ligate these three sizes of cDNA to the vector in different reactions, to obtain representative libraries for all fragment sizes.

### Bioinformatics tools used for *R. microplus* sialotranscriptome analyses

A detailed description of the bioinformatics treatment of the data has been provided elsewhere [21, 23]. Briefly, the ESTs (raw sequences) were trimmed of primer and vector sequences, clustered into contigs (with built-in BLAST [24] and CAP3 assembler [25] algorithms. The contigs were further analysed against several databases, such as the Non-Redundant (NR) protein NCBI database, Gene Ontology (GO) database [26], and the Conserved Domain Database (CDD) [27] containing the KOG [28], Pfam [29], SMART [30] motifs as well as custom-downloaded databases containing the mitochondrial and rRNA nucleotide sequences available at NCBI. A database containing 42,512 ESTs from *R. microplus* available at EST/NCBI database (LIBEST_014697 BEA library; BmiGI database [31]) also was used. We submitted all translated contig sequences to the Signal P server [32] to detect signal peptides indicative of secreted proteins. The counting of ESTs in a contig belonging to each library was used in a chi-square (*χ* ^2^) test to analyse differences in the distribution of ESTs in the contigs, using the chisq.test function in Excel, in which all results with *P* < 0.05 we considered statistically significant. A chi-square test was performed apart for contigs Rm39, Rm180 and Rm239 using SigmaPlot 11.0 (Systat Software, Inc, Sao Jose, CA, USA) to obtained the expected counts of ESTs outputted by the report software. The final output of bioinformatics analyses was piped into a tab-delimited file imported into a hyperlinked Excel spreadsheet (RMallHxN dataset, Table S1, Additional file 1; since the bioinformatics pipeline is built on a Windows system, we advise that Additional file 1: Table S1 is opened from a PC-Windows-based operating system.

### Anti-tick multicomponent vaccine: selection of target genes for molecular cloning and recombinant protein production

Candidate genes for anti-tick vaccines were selected from the comparative transcriptome analysis of ticks fed on susceptible or resistant bovines described herein. The selection criteria included (i) the presence of signal peptide (for saliva secretion) and/or significant blast result with a tick salivary protein previously described; (ii) upregulation in the transcriptome of ticks fed on susceptible bovine compared to resistant hosts; and (iii) important putative function in parasitism, according to BLAST hits in the NR-NCBI database. In addition, for selected candidate genes, epitope predictions were performed using BepiPred method [33] through IEDB-AR (Immune Epitope Database Analysis Resource) [34] for B-cell epitope, as described previously for screening of cattle tick vaccine antigens [35] and TEPITOPE software [36] and NetMHCII 2.2 server [37, 38] for T-cell epitopes**,** both sequence-based methods restricted to HLA-II binding peptides. Target gene sequences were amplified using phage lambda clones from cDNA libraries as templates and specific primers for each gene. The recombinant proteins were produced and named as Rm39, Rm180 and Rm239 (as reference to their contig number in RMallHxH dataset) and all were found to be truncated (partial, incomplete sequence), because the last two, the template phage were not full-length ORF (open reading frame) cDNA clones (5' end sequence corresponding to N-terminus of the protein were missing), and the first one presented the glycine repeats at the C-terminus portion. Both cloning and recombinant protein expression were performed according to the instructions of the Champion™ pET Directional TOPO Expression kit (Life Technologies, Carlsbad, California, USA). Briefly, the PCR products were cloned into the expression vector pET100, and the recombinant His-tagged proteins were produced as insoluble inclusion bodies in *Escherichia coli* BL21Star. The bacteria were harvested and lysed in lysis buffer (8 M urea and 20 mM Tris, pH 8.0). The recombinant proteins were purified in the presence of 8 M (condition for insoluble proteins) urea using HisTrap HP Ni-Sepharose affinity columns (GE Healthcare, Piscataway, New Jersey, USA) according to the manufacturer’s instructions, connected on an AKTA-FPLC system (GE Healthcare, Piscataway, New Jersey, USA). The purified denatured recombinant proteins were refolded as described previously [39] with some modifications. Briefly, the dialysis was performed at 4 °C against 2 l of refolding buffer (25 mM Tris, pH 7.5, 250 mM NaCl, 0.5 mM DTT, 0.1% sodium deoxycholate, 2.5% glycerol and 2–6 M urea) to gradually decrease the urea concentration (three changes, four hours each). Subsequently, the recombinant proteins were dialysed against 0.9% NaCl and checked in 15% polyacrylamide gel electrophoresis (SDS-PAGE). The protein quantification was performed through band densitometry of PAGE using ImageJ [40] to estimate the recombinant protein amount compared to a reference protein (BSA, bovine serum albumina) loaded in known quantities (2 μg, 10 μg and 20 μg). Endotoxin contamination was determined using QCL-1000 Endpoint Chromogenic LAL Assays (Lonza, Walkersville, Maryland, USA). A fourth recombinant protein, named as Rm76 (IgG binding protein C), which was predicted to be putatively glycosylated was expressed (full-length ORF) in F293 mammalian cells (performed by Life Technologies), and also used for the experimental vaccination of cattle. The sequences of antigens were deposited in the ENA/EMBL-EBI database (accession numbers LT795749–LT795752). An SDS-PAGE using 15% polyacrylamide gels and Coomassie blue staining of the *E. coli*-expressed proteins is shown in Fig. S1, Additional file 2. Adding 4 kDa from the expression vector (histidine tag), the molecular weights of the predicted proteins were confirmed.

### Vaccination of cattle and challenge with *R. microplus*

Purebred Holstein calves (3–7-month-old females) were purchased from a tick-free farm and individually housed in stalls with containment fencing at the Institute of Animal Sciences (Nova Odessa, SP, Brazil). The stalls were monitored daily to prevent tick infestation during vaccination to guarantee contact with ticks upon challenge with an artificial infestation. As the calves were of different ages and some of the animals were half-siblings, these animals were arranged based on age and kinship to obtain a varied age group, with minimal genetic background effects.

Purified recombinant proteins in saline (0.9% NaCl) were prepared using an aluminium hydroxide adjuvant at a 1:8 (protein:adjuvant) ratio under sterile conditions. Rm39, Rm180 and Rm239 were prepared, separately, in a mixture containing 100 μg of recombinant protein in a 2 ml dose; Rm76 was prepared as a 25 μg/2 ml dose.

Each calf in the vaccinated group (*n* = 4) was intramuscularly injected in the neck with the four recombinant proteins (in separate injections) using a 3-ml syringe and a 21G needle, three times with 3-weeks intervals (days 0, 21 and 42 of the trial). The calves from the control group (*n* = 4) were injected with saline and adjuvant alone.

At 2 weeks after the last immunisation, the vaccinated and control groups were challenged with 10,000 *R. microplus* larvae in cotton jersey chambers attached on top of the shoulders of the animals (one cell in each body side, infested with 5,000 larvae each). The parasitological parameters of challenge tick infestations were evaluated by counting and weighing the engorged females that spontaneously dropped off at 20, 21 and 22 days of infestation and assessing egg oviposition and egg fertility as previously described [41]. These parameters were used for calculation of vaccine efficacy (E) applying a formula with vaccinated/control group data detailed elsewhere [41, 42]. Briefly, the overall protection (E) conferred through efficient vaccination was calculated based on the effect of the number of ticks (NT, adult female ticks), tick weight (average adult female weight), oviposition (O, average weight of the egg masses per surviving tick), and egg fertility (F, average weight of the larvae per gram of eggs), applying the formula 100 × [1-(CRT × CRO × CRF)], where CRT, CRO and CRF represent the reduction of NT, O and F, respectively, of vaccinated group (V) compared with the adjuvant/saline control group (C), respectively, i.e. CRT = NTV/NTC, CRO = OV/OC and CRF = FV/FC. The tick infestation in one animal from the control group was not considered because the attached cotton cells were damaged, compromising the artificial infestation. The calves were maintained according to the guidelines of the Committee for Ethics in Animal Experimentation of the Ribeirão Preto School of Medicine, University of São Paulo (CETEA-FMRP/USP, certificate numbers 055/2007, 210/2008 and 102/2009). Student’s t-test (*P* < 0.05) was used to compare the results between vaccinated and control groups.

### Detection of antigen-specific antibodies in calf serum

Blood samples were collected from each calf to obtain the sera at different time points: before, after experimental vaccination and during the challenge (larvae infestation). Serum antigen-specific total IgG, IgG1 and IgG2 antibodies were determined through a standard protocol of an indirect ELISA using 0.15 μg/well of purified recombinant Rm39, Rm180, Rm239 and Rm76 sera at 1:50, 1:100 and 1:300 dilutions and incubation with 1:1,000 sheep anti-bovine IgG1 or IgG2-HRP conjugated antibodies (Bethyl Laboratories, Montgomery, Texas, USA), or sera at 1:100, 1:500 and 1:1000 dilutions (for total IgG antigen-specific measurements) and incubation with 1:5,000 rabbit anti-bovine IgG-HRP conjugated secondary antibody (Sigma-Aldrich, St Louis, Missouri, USA). The colour reaction was developed using the TMB Microwell Peroxidase Substrate System (Kierkegaard Perry Labs, Gaithersburg, Maryland). The levels of antigen-specific total IgG and IgG1/IgG2 in the immunised and control calves were expressed as the OD450 value for the 1:500 and 1:100 serum dilutions respectively, and compared between vaccinated and control cattle using ANOVA (*P* < 0.05) and Bonferroni’s *post-hoc* test. The OD450 values for the control group were expressed as the average measurements of all antigens (Rm39, rm239, Rm180 and Rm76). For Western blot analysis, the recombinant proteins (10 μg) were transferred from 12% polyacrylamide SDS-PAGE gels to Hybond ECL nitrocellulose sheets (GE Healthcare, Piscataway, NJ) using a TE 70 semi-dry transfer unit (GE Healthcare). Membranes were incubated with pooled sera (1:100) from non-infested and infested bovines (Holsteins, tick-susceptible breed), collected as previously described [43], or pooled sera (1:100) from vaccinated bovines and sheep anti-bovine IgG1 or IgG2 as secondary antibodies (1:1000). The chemiluminescence detection method was performed using ECL Western Blotting Substrate (Thermo Scientific Pierce, Rockford, Illinois, USA) for horseradish peroxidase (HRP) on an ImageQuant 350 Detection System (GE Healthcare).

## Results

### Characterisation of *R. microplus* cDNA libraries for antigen discovery

It is well known that blood feeding and reproductive efficiency is impaired when ticks feed on resistant host [44]. To ascertain how anti-tick host immunity affects gene expression of *R. microplus* ticks, we analysed eight cDNA libraries of ticks fed on susceptible or resistant hosts (Holsteins and Nelores, respectively). The analysed cDNA libraries were: salivary glands of nymphs, males and females fed on genetically susceptible or resistant hosts (SGNRmS, SGNRmR, SGMRmS, SGMRmR, SGFRmS and SGFRmR).

More than 10,500 clones were randomly sequenced from the eight non-amplified cDNA libraries A total of 7,923 high-quality expressed sequence tags (ESTs) from the eight libraries were obtained, that were assembled into 3,342 unique contigs referred to as the RMallHxN dataset, and functionally annotated (Additional file 1). The distribution of contigs, number of ESTs and singletons were similar for all libraries.

**Fig. 1:**
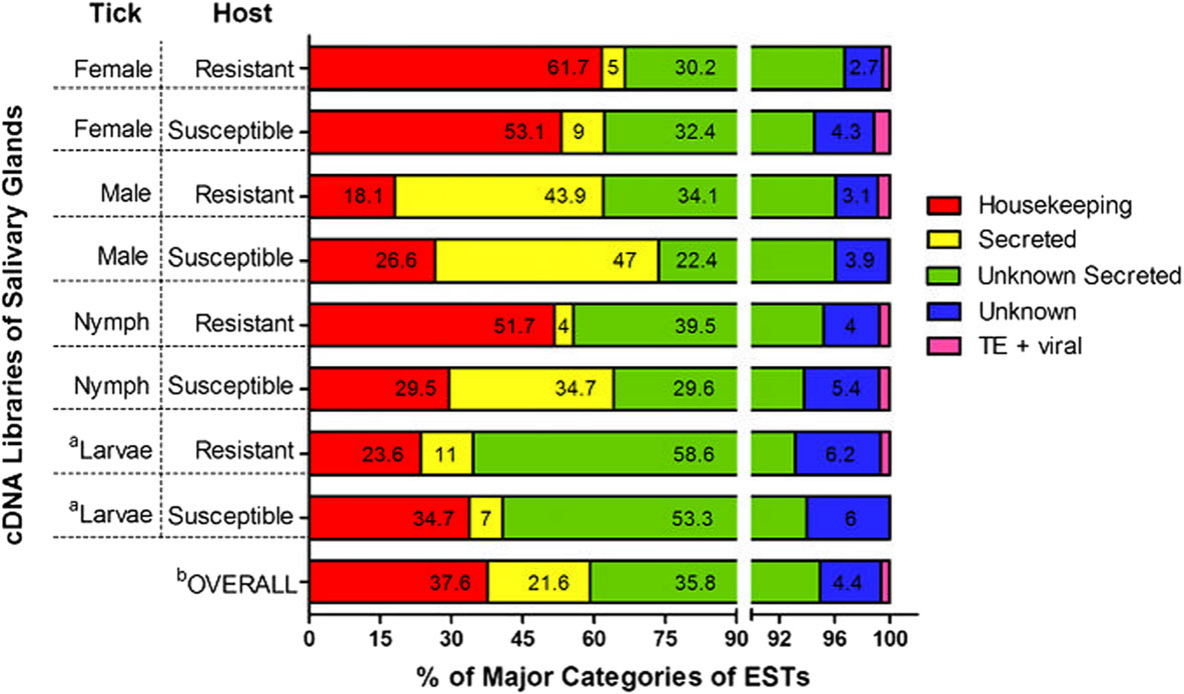
Classification of ESTs from *R. microplus* cDNA libraries. The eight cDNA libraries are represented individually according to the tick’s life stage and which host the tick parasitized. The eight libraries (OVERALL) comprise the RmallHxN dataset. TE: transposable elements. **a** For larvae samples, whole tick crude extract were used to construct cDNA libraries, because salivary glands isolation are not possible for this life stage. **b** The proportions were calculated considering all libraries together rather than separate displayed for eight bars above OVERALL

The ESTs in individual libraries and the combined dataset (RMallHxN) were first grouped into major functional categories: housekeeping, secreted, unknown, unknown secreted, transposable elements (TE) and viral-like (Fig. 1). The proportion of these different categories varying across the libraries primarily depended on the life stage rather than the source of feeding (host), except for nymphs, which presented distinct proportions of housekeeping and secreted transcripts. Interestingly, libraries from male ticks presented the highest proportion of putative secreted proteins with known functions (Fig. 1, ranging from 43.9 to 47.0%), stressing the differences between males, females and nymphs, and suggesting the potential diversity and/or amount of proteins in male saliva. The putative secreted proteins with unknown functions were altered in a similar ratio for females, males and nymphs, comprising more than half of the proportion of the transcripts for larvae libraries. Beyond the quantitative variation, we observed some differences in the transcriptional profile through the manual annotation of the contigs (Additional file 1). Indeed, depending on the source of blood meal, i.e. ticks fed on susceptible or resistant hosts, the expression of many transcripts was altered.

Functional classification of secreted proteins from the RMallHxN dataset (Table 1), revealed that the majority (12.9%) of these proteins belonged to the glycine-rich protein family, which was also previously found to abundantly represented in *R. microplus* [22] and *R. sanguineus* [45] cDNA libraries. This family has a variety of primary functions in ticks involving attachment onto host skin, i.e. fixation, which is essential to ticks start the blood feeding. Expression of glycine-rich proteins has been associated with tick biology (monoxene or heteroxene ticks, and hypostome morphology and size of the cement cone) [22]. Salivary proteins important for haematophagous parasitism, such as proteases (metalloproteases, serine proteases, calpain and serine carboxypeptidases) and proteinase inhibitors (with up to 1% belonging to the Kunitz family), comprised 2.45% of the putative secreted proteins (Table 1).

**Table 1:** Functional classification of putative secreted proteins in the RMallHxN dataset comprising eight libraries (UFLRmS, UFLRmR, SGNRmS, SGNRmR, SGMRmS, SGMRmR, SGFRmS and SGFRmR)

Functional class	Total contigs	Total ESTs	% of total
Related to host immunity^a^	12	23	0.29
TIL domain	16	34	0.43
Proteinase inhibitors			
Kunitz domain	30	87	1.10
Serpin	1	2	0.03
Thyropin domains	1	2	0.03
Cystatin	1	1	0.01
Carboxypeptidase inhibitor	2	3	0.04
Lipocalins/histamine binding proteins	35	61	0.77
Immunoglobulin binding proteins	4	57	0.72
Mucins	13	31	0.39
Basic tail family	10	22	0.28
Enzymes			
Metalloproteases	20	48	0.61
Calpain	6	7	0.09
Serine protease	10	42	0.53
Serine carboxypeptidases	2	2	0.03
Antigen 5 family	4	23	0.29
Glycine rich family			
Cement proteins	112	1024	12.92
GYY family	15	70	0.88
GGY family	4	8	0.10
Other secreted proteins	12	13	0.16
*R. microplus*-specific proteins	107	296	3.74
Unknown secreted	2,082	2,836	35.79

^a^These transcripts encoding proteins that may play role in modulation of host immunity. They were classified as “similar to protein associated with interferon”, “microplosin family”, “DAP-36 immunosuppressant family”, “Evasin”, “Defensin” and “Ixoderin” as displayed on Additional file 1

To determine the novelty rate of the RMallHxN dataset in comparison with the *R. microplus* ESTs database, we blasted the 3,342 contigs in the RMallHxN dataset against the BmiGI database, a database of *R. microplus* ESTs published by Guerrero and colleagues, obtained from a cDNA library (called LIBEST_014697 BEA available at EST/NCBI database) of different tissues and life stages of *R. microplus* [46]. It contains 45,512 ESTs resulting in a total of 13,643 unique transcripts [46]. Notably, 47.2% of the contigs in the RMallHxN dataset has no BLAST hits against the BmiGI database (Additional file 1, column AW, “Best match to BEA database”). Many factors may have influenced in this high level of novelty observed in our transcriptome, such as the strategy employed for the construction of the cDNA libraries, starting with the mRNA from target tissue (salivary glands) of ticks feeding on resistant hosts and, most importantly, the contig assembling parameters employed by our bioinformatics pipeline.

### Selection and production of tick salivary gland antigens

The analysis of the comparative transcriptome from *R. microplus* ticks fed on susceptible or resistant hosts (RMallHxN dataset) generated a catalogue of transcripts that were differentially expressed according to the origin of the blood meal, and this catalogue was used to select potential candidates for the development of a cattle tick vaccine. We applied the following criteria to select genes for evaluation as potential vaccine antigens: (i) the presence of a signal peptide, indicating secretion in saliva or significant blast result with tick secreted salivary protein described previously; (ii) important putative function for haematophagous parasitism; and (iii) upregulation of expression in ticks feeding on a susceptible host. Because biochemical functional characterization studies for *R. microplus* salivary gland proteins are scarce, the second criterion was based on the blast result with proteins that probably play a role in haematophagy, i.e. sequence similarities with proteins predicted by other sialotranscriptome studies to be involved in coagulation, immunosuppression, anti-inflammatory responses and tick attachment.

Based on these criteria, we listed a few dozen of antigen candidates from the RMallHxN dataset (Additional file 1) to be tested as vaccine targets. Here, we described the first trial of an immunisation test, in which 11 priority candidates (all with putative function in haemostasis, host immunomodulation and tick attachment) were chosen to start the procedures of PCR amplification, cloning and protein production. We successfully cloned and expressed three N-terminal-truncated recombinant salivary proteins, Rm39, Rm180 and Rm239, in an *Escherichia coli* system. The transcripts encoding Rm39, Rm180, and Rm239 showed similarity to glycine-rich proteins, serine protease inhibitors and metalloproteases, respectively and all of these transcripts were significantly increased in ticks fed on susceptible hosts (Table 2). The cloned sequences encode N-terminal histidine-tagged proteins of 54, 67 and 145 amino acids corresponding to theoretical molecular weights of 5.8, 7.9 and 16.5 kDa, respectively (Table 2). The phage clones for Rm180 and Rm239 amenable to cloning procedures were not full-length sequences, in which portions of 5' end sequence for both were missing, therefore the recombinant proteins were produced as N-terminal truncated. The full-length sequences for Rm180 and Rm239 were obtained further with a high-resolution sialotranscriptome obtained by next-generation sequencing (unpublished data, NCBI BioProject PRJNA329522). Although the contig Rm39 presented a full-length sequence (Additional file 1), we cloned only the 54 amino acids at the C-terminal portion, where repeats containing glycines are located.

**Table 2:** Characteristics of candidate antigens from the *R. microplus* transcriptome selected using reverse vaccinology for assessment in an anti-tick vaccine

Transcripts from cDNA libraries (RMallHxN dataset)							Recombinant protein			Full-length sequence^e^		
	Developmental stage	No. ESTs in RmR^a^	Expected No. ESTs in RmR^j^	No. ESTs in RmS^b^	Expected No. ESTs in RmS ^j^	*P* value^c^		Amino acids	MW^d^ (kDa)		Amino acids	MW (kDa)
Rm39	All stages	21	30	44	34	0.033^f^	Rm39	54	5.8	Rm39	105	11
Rm180	Female, Nymph, Male	5	14	27	18	< 0.001^g^	Rm180	67	7.9	Rm180	186	20.8
Rm239	Nymph, Male	7	18	26	14	0.007^h^	Rm239	145	16.5	Rm239	500	55

^a^ *R. microplus* ticks fed on resistant hosts

^b^ *R. microplus* ticks fed on susceptible hosts

^c^Chi-square test using SigmaPlot 11.0 software

^d^Molecular weight calculated without a histidine tag. The size of tagged recombinant proteins is shown in Additional file 2: Figure S1

^e^Complete sequences of antigens

^f^Chi-square test, *χ* ^2^ = 4.531, *df* = 1

^g^Chi-square test, *χ* ^2^ = 10.864, *df* = 1

^h^Chi-square test, *χ* ^2^ = 7.343, *df* = 1

^j^Expected counts were obtained from software report

As Rm76 and Rm239 vaccine candidates are larger than Rm180 and Rm39, they probably contain more antigenic determinants. Then, we analysed these selected antigens regarding their immunogenicity using *in silico* epitope prediction methods for both T-cell and B-cell epitopes. T cell epitope prediction was restricted to MHC class II because we believe that the antibody response helped by antigen-specific CD4+ T cells will be the protective immunological mechanism elicited by an efficacious tick vaccine, in which the specific antibodies produced against saliva components neutralise the action of these tick proteins on hosts during the hematophagy. Since there is no available tool to predict BoLA class II binding peptides, we used resources available for human alleles, such as TEPITOPE and NetMHCII.

As shown in Table 3, Rm239 and Rm76 antigens were richer in peptides that bind MHC class II alleles as well as richer in peptides that bind to a broader range of MHC class II alleles. When we considered the native antigen (complete protein sequence), Rm239 presented the most numerous T-cell epitopes, which can be partly explained by its long protein sequence. Despite the similar lengths of Rm180 and Rm76 native antigens, the latter presented eight times more epitopes. Rm39 presented the lowest numbers of binding peptides. Similar epitope prediction profiles were observed with both tools (Table 3), i.e. Rm76 and Rm239 presented more MHC class II binding peptides.

**Table 3:** Epitope prediction of HLA-II-restrict T cells using Rm39, Rm180, Rm239 and Rm76 protein sequences with TEPITOPE and NetMHCII software

TEPITOPE			
Native antigen (full-length sequence)	No. of peptides^a^	No. of alleles^b^	Score^c^
Rm239 (500 amino acids)	2,023	54	31.22
Rm76 (152 amino acids)	867	54	30.85
Rm180 (186 amino acids)	105	53	43.27
Rm39 (105 amino acids)	38	34	4.05
Recombinant vaccine antigen^d^ (partial sequence)	Position^f^	No. of alleles^b^	Score^g^
Rm180	25	53	24.9
Rm39	69	5	0.6
	30	9	2.3
Rm239	79	2	0.23
	59	27	3.06
	38	5	1.30
Rm76^e^	157	52	24.5
	137	52	9.7
	132	52	2.4
	129	53	6.2
	122	53	25.7
	121	53	17.8
	120	52	7.5
	117	54	5.7
	116	53	5.0
	66	52	15.0
	62	35	3.3
	56	53	22.1
	50	1	1.2
	46	53	11.9
	40	53	5.0
	39	53	15.7
	37	3	2.4
	36	53	12.0
NetMHCII			
Recombinant vaccine antigen^e^	No of peptides^h^	No of Alleles^i^	Strong ligands^j^
Rm180	52	10	33
Rm39	72	6	–
Rm239	130	14	75
Rm76 ^e1^	138	14	166

^a^Total number of epitopes with positive score

^b^A total of 54 HLA-DR alleles were used by TEPITOPE. The values refer to the number of HLA-DRB alleles that binds to predicted epitopes

^c^The score (ranging from negative to positive values) is a calculated value to define the binding affinity between peptide and HLA, therefore the higher the score, the higher the binding affinity. The values are expressed as the mean of scores from predicted bindings

^d^Data from sequences (partial) of recombinant proteins used in this study

^e^Rm76 was the only antigen cloned as full-length sequence

^f^Position of the first amino acid of the HLA class II binding peptide on the protein sequence

^g^Only the positive scores were considered, i.e. those with the most binding affinity properties

^h^Total number of possible peptides based on each protein size

^i^A total of 14 HLA-DR alleles were used by NetMHCII v. 2.2. The values refer to the number of alleles that presents predicted ligands

^j^The binding affinity to HLA-DR is based on IC_50_ values in nM. The threshold for strong ligands is IC_50_ = 50. Only results for strong ligands are shown

**Fig. 2:**
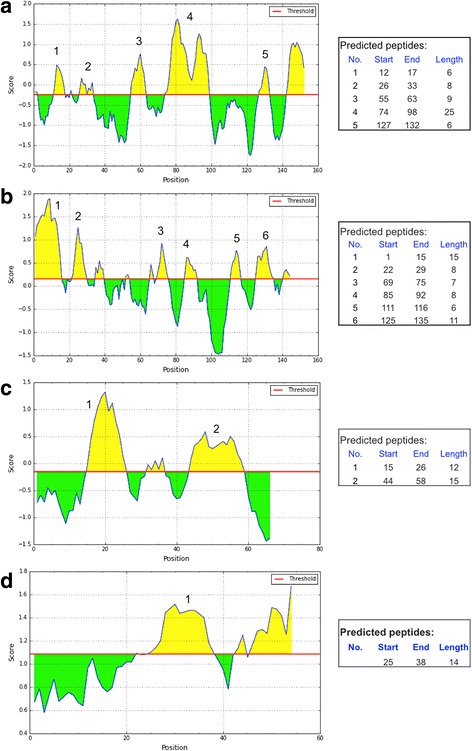
B-cell epitope predictions. The sequences for recombinant salivary proteins Rm76 (**a**), Rm239 (**b**), Rm180 (**c**) and Rm39 (**d**) were used to predict linear B-cell epitopes with BepiPred method through IEDB-AR web-based repository. The red line represents the threshold value to assign epitope sites across amino acids position in protein sequences. Only predicted peptides longer than six amino acids were depicted

To predict B-cell epitopes, we used BepiPred that combines a hidden Markov model and a propensity scale method (Parker Hydrophilicity [47]) to identify potential sites in protein (linear peptides highly prone to be exposed on native protein surface) that are recognised by antibodies. As observed for T-cell epitope prediction, Rm76 and Rm239 were predicted to present more antigenic determinants (Fig. 2a, b, respectively) than Rm180 and Rm39 (Fig. 2c, d, respectively). Together, these epitope predictions suggested that Rm76 and Rm239 might elicit a better antibody repertoire.

We were unable to confirm whether these tick salivary antigens are consistently produced in saliva during a blood meal or whether they are secreted on a specific day of feeding (e.g. using Western blot of tick saliva with anti-Rm39 or anti-Rm239 or anti-Rm180 antibodies). However, we gathered indirect confirmation that at least Rm239 and Rm76 (or very closely related proteins) are secreted in saliva because we observed an elevation in antibody titres for these antigens after the challenge infestation, i.e. tick bites (with inoculation of saliva; described in the next section).

### Vaccination of calves with the tick recombinant salivary proteins

The candidate antigens to be evaluated in this study are predicted to function at the tick-host interface because they presented as upregulated transcripts when *R. microplus* ticks feed on susceptible hosts. Thus, we propose that bovines infested with ticks could develop an antibody response to neutralise the effects of these salivary antigens during infestation provided they are properly delivered to the host.

To evaluate the capacity of the selected antigens to induce protection against tick infestations, we immunised Holstein calves with the recombinant *R. microplus* salivary proteins Rm39, Rm76, Rm180 and Rm239. The vaccinated group received three separate doses of the four recombinant antigens at 3-week intervals, followed by challenge with *R. microplus* larvae at 2 weeks after the last immunisation.

We observed that the feeding time on ticks fed on vaccinated animals was slowed and the ticks did not present the typical aspect of fully engorged females by the 22nd day of challenge infestation. This was reflected by the significant reduction in the number of female ticks (52.5%; t-test, *t*_(3.292)_, *df* = 3, *P* < 0.0460) and tick engorgement weight (55.2%; t-test, *t*_(2.830)_, *df* = 5, *P* < 0.0367) that were observed in vaccinated calves (Table 4) when compared to calves that received adjuvant alone. The weight of egg masses and their hatching rates tended to be smaller in vaccinated animals relative to the same parameters observed in ticks fed on control animals. However, the differences were not found to be statistically significant. Vaccination with recombinant salivary antigens induced an overall protection of 73.2% in the vaccinated calves, according to the formula for calculations of vaccine efficacy (Table 4). Because tick numbers and tick weights were significantly reduced, we conclude that some or all of the salivary antigens tested were able to control tick infestations in vaccinated animals.

**Table 4:** Control of *R. microplus* infestation in cattle vaccinated with recombinant salivary antigens selected from the comparative transcriptome of ticks fed on susceptible or resistant hosts

	Parameters of parasitism				
Experimental group^a^	Percent reduction in tick burden (no. of adult females)^b^	Percent reduction in tick weight (adult female weight in mg)^b^	Percent reduction in oviposition (egg masses weight in mg per survived tick)^b^	Percent reduction in egg fertility (larvae weight in grams of per gram of eggs)^b^	Efficacy (%)^c^
Vaccinated	52.5 (587 ± 189)^**^	55.2 (151 ± 51)^**^	18% (88 ± 20)	27.2 (0.016 ± 0.006)	72
Adjuvant/saline control	1,233 ± 51	338 ± 29	107.5 ± 8.7	0.022 ± 0.014	–

After 2 weeks of last immunization dose calves were challenged with 10,000 larvae. Because *R. microplus* is a monoxenic tick, infestations are evaluated by counting of engorged females and their reproductive efficiency

^a^Holstein calves with kinship were separated in different groups (vaccinated group, *n* = 4; control group, *n* = 3)

^b^Percent reduction was determined in relation to the control group. Mean ± SE values are in parentheses. Student’s t-test was used to compare vaccinated and control groups (**P < 0.05)

^c^Vaccine efficacy based on the reduction in the number of female ticks (CRT), oviposition (CRO) and egg fertility (CRF) compared with the control group using the formula 100 [1-(CRT × CRO × CRF)]

**Fig. 3:**
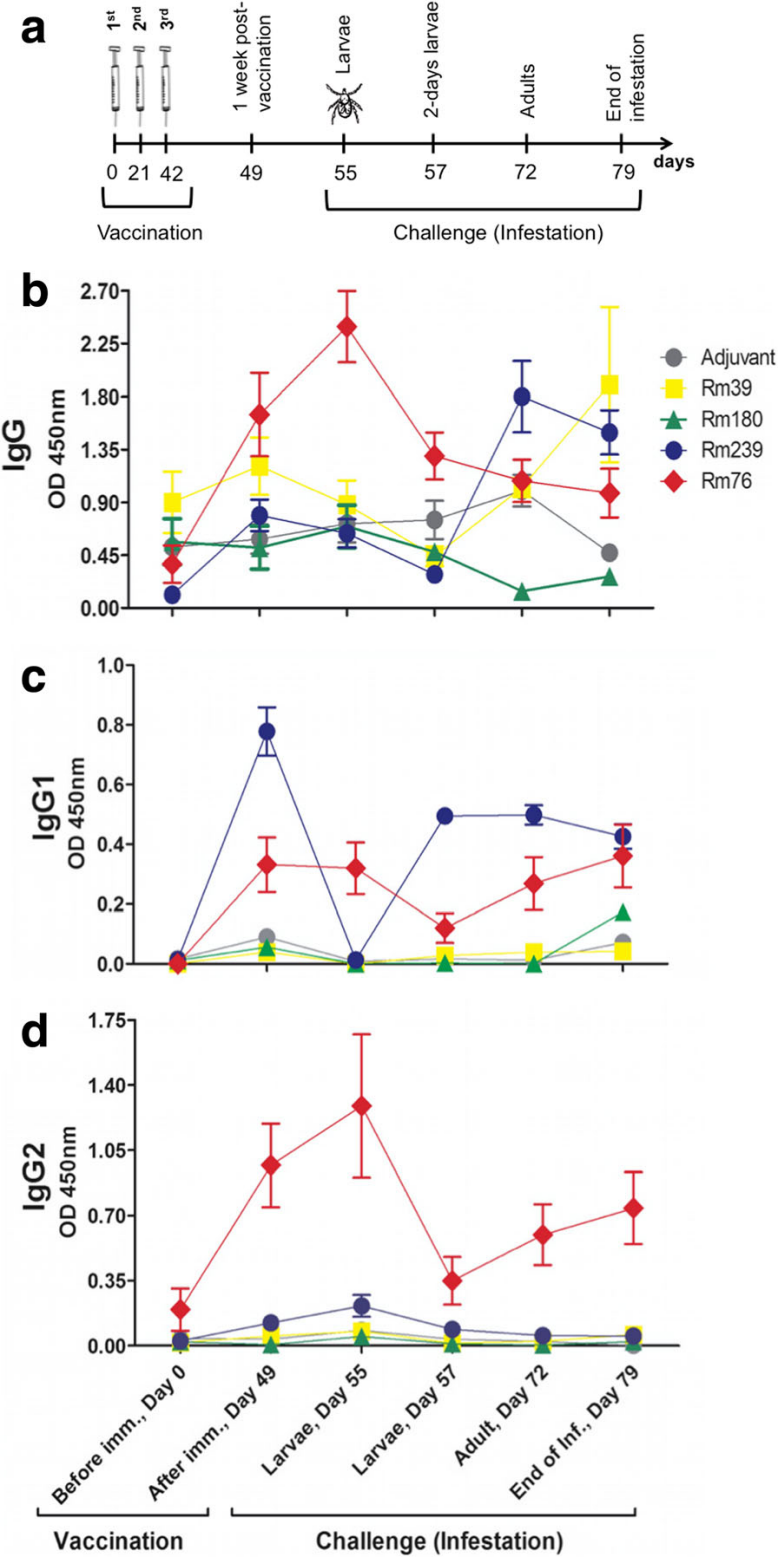
Antigen-specific IgG (total), IgG1 and IgG2 antibody responses upon vaccination and challenge with *R. microplus*. **a** Schematic representation of vaccination trial indicating the measurement periods of antigen-specific antibody responses. **b** Indirect ELISA was used to evaluate total IgG and its subclasses, IgG1 (**c**) and IgG2 (**d**) antigen-specific antibody responses in sera from vaccinated and adjuvant control bovines. The data represented the average of duplicate values at 1:500 dilution for total IgG and 1:100 dilutions for IgG subtypes

The antigen-specific antibody response was evaluated in both experimental groups according to the measurements of total IgG, IgG1 and IgG2 antigen-specific antibodies in the sera of the calves before and after the vaccination (1 week after the third injection) and also upon challenge with *R. microplus* ticks (Fig. 3). The measurements for Rm76 showed that a significant induction of anti-Rm76 antibodies was elicited after vaccination, with a major contribution of the IgG2 subclass (Fig. 3d). Despite not being significant, the levels of total IgG anti-Rm239 also presented a small increase after the vaccination. Interestingly, a significant increase in the levels for total IgG anti-Rm239 antibodies was reached during challenge infestation (Day 72, adult infestation period, Fig. 3b, c), most likely due to a major contribution of IgG1 subtype response took place (Fig. 3c). In general, we observed that the Rm239 and Rm76 antigens induced a significant antibody response in vaccinated animals for IgG1 and IgG2 subtypes (ANOVA, *F*_(1.521, 44)_ = 6.234, *P* < 0.001; *F*_(3.299, 44)_ = 22.09, *P* < 0.001; Fig. 3c, d, respectively).

The antigen-specific IgG1 response induced by Rm239 antigen after vaccination (Fig. 3c, Day 49) was drastically reduced 1 week later (Fig. 3c, Day 55). The half-life of IgG1 is shorter than IgG2 [48], a fact that might explain this finding. Noteworthy, however, is the early recall production of anti-Rm239 IgG1 antibodies 2 days after challenge with *R. microplus* larvae exhibited by the vaccinated calves (Fig. 3c, Day 57), a response that persisted until the adult life stage of the tick (Fig. 3c, Day 72) and began decreasing at the end of infestation (Fig. 3c, Day 79). Future studies should observe antibody titers after the second infestation of cattle.

The host response against Rm76 exhibited significant levels of specific IgG2 when compared with the adjuvant control group, reaching peak levels 1 week after vaccination (Fig. 3d, Day 49). Moreover, at day two after challenge with *R. microplus* (Fig. 3d, Day 57), levels of anti-Rm76 IgG2 were considerably reduced and then subsequently increased during infestation at the adult tick stage (Fig. 3d, Day 72). The reduction in the antibody response observed 2 days after challenge (Fig. 3d, Day 57) might be due to the immunomodulatory effects of tick infestation, through the several immunosuppressant molecules in tick saliva [44, 49], in addition to the modulatory immune responses mounted by susceptible hosts themselves, such as the acute phase protein haptoglobin, which rises during infestations [50]. Haptoglobin presents a negative effect on the immune system [51], including the inhibition of the antibody response to T-cell dependent antigens and immunoglobulin synthesis [52].

The results obtained for the Rm239 and Rm76 antigens suggested that these salivary proteins are produced and secreted into the saliva of ticks during blood feeding, as both proteins stimulated a specific recall antibody response during the post-challenge period (specific IgG1 anti-Rm239 and anti-Rm76 and specific anti-Rm76 IgG2). Levels of both anti-Rm76 IgG1 and IgG2 antibodies were low during the larval stage of infestation and increased at the adult stage. With the depth of sequencing reached in this study the sialotranscriptome analyses showed that Rm239 was expressed in nymphs and male ticks (Table 2). Future studies using techniques such as quantitative PCR and Western blotting should be done to determine if Rm239 is also expressed and produced in larvae and adult salivary gland tissues. Antigen-specific antibody production and the results of the sialotranscriptome analyses are consistent with the production of Rm76 in male ticks (Additional file 1). In summary, Rm239 and Rm76 were demonstrated to be most suitable antigens to naturally boost the immune response in vaccinated animals. Antibody titres against Rm76 and Rm239 antigens (total IgG, IgG1 and IgG2) are available in Additional file 3: Figure S2.

**Fig. 4:**
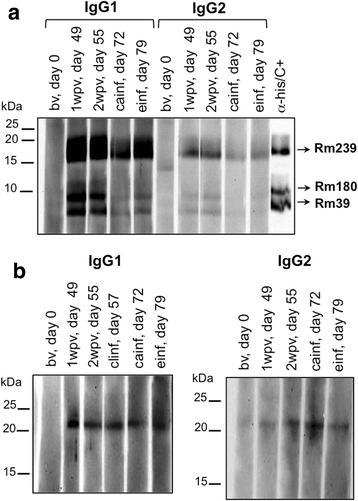
Antigen-specific antibody recognition for denatured Rm39, Rm180, Rm239 and Rm76 antigens. Western blot analysis of the recombinant salivary proteins Rm39, Rm180, Rm239 (**a**) and Rm76 (**b**) using pooled sera (1:100) from vaccinated calves for the evaluation of antibody responses to linear epitopes. *Abbreviations*: Bv, before vaccination; 1wpv, 1 week post-third shot of vaccination; 2wpv, 2 weeks post-third shot of vaccination; cainf, challenge infestation (bovines infested with ticks in adult stage); clinf, challenge infestation (bovines infested with ticks in larvae stage); einf, end of infestation

Unexpectedly, specific IgG subtype (IgG1/IgG2) responses against Rm39 and Rm180 were not detected by ELISA (Fig. 3). Despite not being statistically significant, an increase of total IgG anti-Rm39 was observed at the end of infestation, while the total IgG anti-Rm180 remained detectable at low levels. Thus, we performed SDS-PAGE followed by immunoblotting for Rm39, Rm180 and Rm239 using the pooled sera from vaccinated and control calves to evaluate the antibody response to a second method. Analysis of the antibody recognition patterns of Rm39, Rm180 and Rm239 proteins in vaccinated animals through Western blot revealed that the animals from the vaccinated group produced IgG1 antibodies against the three recombinant salivary proteins at 7 and 14 days after the third immunisation, but that the tick infestation did not stimulate the IgG1 recall response for the Rm39 and Rm180 antigens (Fig. 4a). The same recognition pattern was observed for IgG2 antibodies but at a much lower intensity. The pooled sera obtained from the vaccinated group did not recognise the recombinant salivary antigens before vaccination, as expected (Fig. 4a, “bv” lanes), and similar results were obtained for the pooled sera obtained from the control group (data not shown).

We also performed immunoblotting for Rm76 using pooled sera obtained from the vaccinated and control groups. The animals produced IgG1 antibodies after vaccination and during tick infestation (Fig. 4b). IgG2 antibody production was delayed 1 week compared with IgG1, and IgG2 antibodies were also detected against Rm76 at 14 days after vaccination and during tick infestation (Fig. 4b). The immunoblotting results of Rm239 and Rm76 corroborated the observations in ELISA measurements. Through Western blot we detected a specific antibody response (the majority being of the IgG1 subtype) for Rm39 and Rm180. However, these antibodies were not strongly elicited during challenge tick infestations, as observed for Rm239 and Rm76 antigens. Furthermore, the antibody responses were in line with the findings for epitope predictions.

## Discussion

William Trager demonstrated more than seven decades ago that immune responses are induced against ticks through the bites they inflict during repeated infestations [53] that these responses could be passively transferred to naïve hosts by serum. These host responses resulted in damage to feeding ticks and reduced acquisition of blood by female ticks during subsequent infestations on both actively and passively immune hosts [53] and validated the notion that stimulation of immunity against tick proteins using vaccines is an adequate and sustainable alternative to control infestations. Trager also showed that salivary glands were the most efficient tissue with which to induce anti-tick immunity. However, the remarkable results above were described for unnatural tick-host associations (*Dermacentor variabilis* and Guinea pigs), but Trager described in the same paper that anti-tick immunity does not develop as promptly in natural tick-host associations (*D. variabilis* and *Peromyscus leucopus*), perhaps because the specialized tick saliva can selectively block their hosts’ immunity and inflammation [54]. Much later, Brown et al. [55] and Shapiro et al. [56] showed that immunisations of hosts with fractions of tick saliva induced similar detrimental effects upon a tick’s life-cycle. In spite of this early success, and perhaps due to the difficulties posed by natural tick-host associations, antigen selection for development of bovine anti-tick vaccines was subsequently diverted from testing salivary antigens towards a preference for “hidden” or “concealed” tick antigens, thus called because the host is not exposed to them during natural infestations and, consequently, the tick would not have developed an escape mechanism for these potential vaccine targets. All of the commercially available anti-tick vaccines were developed based on this principle, but they present variable efficacies and still require the application of acaricides for tick control [57]. Thus, development of an effective anti-tick vaccine is still in demand. The premise of the approach adopted in the present work is that the vaccine antigens should be involved in important biological processes for parasitism, i.e. salivary antigens. Furthermore, memory can be boosted since vaccinated hosts will be exposed to tick saliva during challenges. Lastly, the vaccine must comprise a multi-component formulation to decrease variations in vaccine performance due to genetic diversity in bovines and tick strains and also to cover the main functions of saliva in all stages of parasitism. Importantly, this strategy can affect various stages of the tick’s life-cycle resulting in decreases in tick populations to levels better tolerated by cattle.

Our results show that mining the sialotranscriptome and larvae transcriptome based on differential expression modulated by host anti-tick immunity is helpful for the identification of protective vaccine antigens. Rodriguez-Valle et al. [58] were the first to evaluate if host anti-tick immunity affects transcript expression patterns when ticks fed on *Bos indicus* (tick-resistant) or *Bos taurus* (tick-susceptible) cattle. For this, they employed a microarray based on the BmiGI database [31], which contains transcripts of ticks fed on *Bos taurus* hosts. With this approach, they demonstrated that the genetic composition of bovine hosts changes the gene expression profile in ticks during blood meals. Besides corroborating these findings by Rodriguez-Valle et al. the present study generated a transcriptome using a sequencing-based approach, which provides not only the expression pattern (up- or downregulation), but also enables the identification of transcripts exclusively expressed in one condition.

In this first trial, described herein, we selected some candidates and were able to produce successfully four recombinant antigens and tested them as multicomponent tick vaccine. Vaccinated animals were able to control infestations using the reduction of tick burden and tick engorgement. Future studies in a larger number of animals, immunised with a larger diversity of antigens, assessment of dose regimens and also formulation with other adjuvants should be undertaken to improve the outcomes observed using this strategy.

An approach of similar thinking has been applied to antigen discovery for vaccine-based prevention of the schistosomiases by screening protein libraries with sera from schistosomiasis-resistant and chronically infected humans [59]. Herein, we selected gene candidates for immunisation of bovines genetically susceptible to infestation with a multi-component vaccine. After challenge with *R. microplus* larvae, the tick infestation was significantly controlled in the vaccinated bovines, showing 73.2% vaccine efficacy. Additionally, we have evaluated the gene expression profiles elicited by this recombinant vaccine to ascertain molecular signatures; this analysis will provide more details about the response of the host to vaccination and infestation.

Large-scale searches for protective antigens against ixodidae ticks has been performed such as for *Ixodes* [60, 61], and *R. microplus* species [62, 63]. However, the screening strategies either target tissues that do not mediate parasitism directly (e.g. cultured IDE8 embryonic *I. scapularis* cells) and/or performed large-scale screening of expression libraries with sera from infested hosts, which will not necessarily recognise all the useful targets in saliva. Indeed, the salivary antigens tested in the present work were silent antigens and did not readily elicit immune responses in Holstein bovines successively infested with ticks (data not shown). In addition, none of these antigens target candidates whose expression and production was affected through the natural immunity of the host. The differential expression of salivary transcripts in response to host immunity associated with the putative function of the proteins provided insight into the important antigens involved at the tick/host interface. Other differential transcriptome data [14] of ticks fed on resistant or susceptible bovines were obtained using a customised microarray based on the BmiGI database, i.e. it is represented only by transcripts from ticks fed on a susceptible host and, furthermore, without tissue-specific resolution. Our data shows that several transcripts are expressed only in ticks feeding on resistant hosts (see Additional file 1).

Many of the antigens evaluated as anti-tick vaccines in cattle, i.e. embryo proteins [64], ferritin 2 [42], subolesin and ubiquitin [62], 5'-nucleotidase [65], Bm95 [66, 67], and the Bm86 antigen from commercial vaccines [4], are concealed antigens. Although vaccination with these antigens elicits an antibody response [68], these proteins are not exposed to the hosts through tick bites, and the memory immune responses against them are not stimulated through saliva as a natural boost in immunity against ectoparasites. Alternatively, to the extent that some of these intracellular proteins are secreted to the host perhaps by a holocrine mode, these vaccines may be effective, but proof of secretion in this non-canonical form is missing for the majority of targets, except for the tick histamine release factor [69]. Therefore, vaccines that use only concealed antigens require several booster injections for the maintenance of immunity in vaccinated animals, which is expensive and difficult to manage in livestock.

Recent trials with similar goals deserve special comment: they used the candidate gene approach and tested single proteins from *R. microplus* in cattle. The function of one of these antigens [70], a metalloprotease, is similar to one antigen tested in this study, however, it afforded lower protection, and one of four animals did not respond, supporting the need for a cocktail vaccine. Another antigen, an aquaporin derived from tick gut [71], afforded a level of efficacy similar to this study’s vaccine, but challenge infestations did not boost the level of antibodies foreboding poor memory, similar to what occurs with commercial vaccines, which employ gut antigens. A third trial employed a salivary antigen, acidic ribosomal protein P0, conjugated to keyhole limpet haemocyanin (KLH) that afforded an efficacy of 96% [72]. However, that study did not ascertain if the carrier induced cross-reactivity to tick haemocyanins and the antibody responses to the antigen were variable and boosts by the challenge cannot be established as well.

In our initial vaccination trial using a multi-component vaccine containing four recombinant salivary antigens, we observed that specific antibody production was efficiently stimulated against two antigens, Rm239 and Rm76, after immunisation and during infestation with *R. microplus*. The Rm239 and Rm76 salivary antigens possess putative metalloprotease and IgG binding functions, respectively. The neutralisation of these salivary proteins during tick infestation through specific IgG subtypes antibodies might represent one of the mechanisms involved in protection against ticks. Impairing important salivary proteins can be effective for the control of tick infestations. Notably, the dynamics of reactivity of IgG1 with those antigens was complex, whereas IgG2 displayed more uniform patterns of reactivity with Rm239 and Rm76. Those results suggest that salivary antigen-specific IgG exhibit varied half-lives, whereby several factors could influence their degradation and clearance as: the subclass [48], amino acid composition of Fab regions determined by specificity [73] and patterns of glycosylation [74, 75]. Importantly, these two recombinant salivary antigens, Rm239 and Rm76, indicated that tick saliva elicits a recall antibody response, even during the early stages of infestation, and possibly elicits the protection observed in the vaccinated group, consistent with the results obtained herein.

Conversely, the recombinant salivary antigens Rm39 and Rm180 maybe induced a small repertoire of specific antibodies, primarily comprising IgG1, which recognised epitopes after immunisation, but not during the first infestation. They presented lower immunogenicity than Rm76 and Rm239, a finding that was subsequently confirmed by B-cell and T-cell epitope prediction analyses. Further studies testing successive challenge infestations should be done to ascertain if repetitive infestation will boost the immune responses to weakly immunogenic Rm39 and Rm180 antigens. Also, modifications in vaccine formulations for the Rm39 and Rm180 antigens should be performed to increase the antibody response stimulated through immunisation. These salivary proteins should be further studied and considered as potential antigens because these proteins might be involved in mechanisms that underlie the tick blood feeding process. Since Rm180 salivary antigens possess putative serine protease inhibitor functions, these proteins could disturb certain host homoeostasis processes involving serine proteases. The Rm39 salivary antigen shares similarities with extracellular matrix proteins and this protein could act as a decoy for the host. Accordingly, these antigens might play important roles in subversion mechanisms to evade host immune responses directed against tick salivary proteins, as parasites present a wide range of mechanisms to escape from host defences, including haematophagous parasites, such as ticks [76]. Interestingly, the scores obtained in the TEPITOPE and NetMHVII v2.2 T cell epitope prediction algorithms for Rm39 and Rm180 antigens indicated that they present fewer T cell epitopes than the Rm239 and Rm76 antigens. These algorithms employ HLA alleles; however other authors have shown that they can also predict the immunogenicity of antigens for bovines [77, 78].

In summary, the results reported herein show that our approach is promising as a strategy to identify new cattle tick vaccine antigens. The effective control of tick infestations through immunological interventions as vaccines still depends on the discovery and evaluation of other antigens. In addition, the formulation of a multi-component anti-tick vaccine comprising both types of antigens, exposed and concealed, might be more helpful by decreasing the effects of cattle genetic diversity, providing a natural boost through the saliva and escaping the selective pressure of the host.

## Conclusions

This work is significant for antigen discovery because our strategy applies to other parasites. It is also significant for scientists working on tick vaccines, for producers affected by tick infestations and for consumers concerned about contamination of environment and food products with acaricides. Here we show that tick sialotranscriptome analyses guided by the immunity of tick-resistant hosts selected important vaccine targets and that tick vaccine targeting a range of immunogenic tick salivary proteins weaken parasitism, boost immune memory and can achieve sustainable tick control.

## Additional files

Additional file 1: Annotated sialotranscriptome (RMallHxN dataset) of the *Rhipicephalus microplus* tick. (.docx) available at http://exon.niaid.nih.gov/transcriptome/Rhip_microplus/2015-07/Table_S1.zip. (DOCX 25 kb)

Additional file 2: Figure S1. Recombinant salivary proteins of *Rhipicephalus microplus*. Partial sequences of the selected candidates Rm39, Rm180 and Rm239 were expressed in *E. coli*. SDS-PAGE using 15% polyacrylamide gels and Coomassie blue staining (.tiff). Adding 4 kDa from the expression vector (histidine tag), the molecular weights of the predicted proteins were confirmed. (TIF 115 kb)

Additional file 3: Figure S2. Antigen-specific antibody titres for Rm239 and Rm76 antigens. (DOCX 310 kb)

## Abbreviations

CDD: Conserved Domain Database
cDNA: Complementary deoxyribonucleic acid
DTT: Dithiothreitol
E: Percentage of vaccine efficacy
ECL: Enhanced chemiluminescence
ELISA: Enzyme-linked immunosorbent assay
ESTs: Expressed sequence tags
Fab: Fragment antigen-binding
GO: Gene ontology
HLA-II: Human leukocyte antigen class II
HRP: Horseradish peroxidase
IEDB-AR: Immune Epitope Database Analysis Resource
IgG: Immunoglobulin G
KLH: Keyhole limpet hemocyanin
NR: Non-redundant
ORF: Open reading frame
PAGE: Polyacrylamide gel electrophoresis
PBS: Phosphate buffered saline
SGFRmR: Salivary glands of females fed on resistant host
SGFRmS: Salivary glands of females fed on susceptible host
SGMRmR: Salivary glands of males fed on resistant host
SGMRmS: Salivary glands of males fed on susceptible host
SGNRmR: Salivary glands of nymphs fed on resistant host
SGNRmS: Salivary glands of nymphs fed on susceptible host
signal P: Signal peptide
TE: Transposable elements
TMB: 3,3',5,5'-Tetramethylbenzidine
UFL: Unfed larvae
UFLRmR: Unfed larvae hatched by females fed on resistant host
UFLRmS: Unfed larvae hatched by females fed on susceptible host
V: Vaccinated group
