# Supplementary material for: Mining a differential sialotranscriptome of Rhipicephalus microplus guides antigen discovery to formulate a vaccine that reduces tick infestations
Source: Parasit Vectors. 2017 Apr 26;10:206. doi: 10.1186/s13071-017-2136-2 (PMC5406933; doi:10.1186/s13071-017-2136-2)
Supplement: Supplementary file 1 — Annotated sialotranscriptome (RMallHxN dataset) of the Rhipicephalus microplus tick. (.docx) available at http://exon.niaid.nih.gov/transcriptome/Rhip_microplus/2015-07/Table_S1.zip. (DOCX 25 kb) [file 13071_2017_2136_MOESM1_ESM.docx]

**Additional File AF1 1.** RMallHxN dataset: annotated catalogue of transcripts expressed in larvae and salivary glands from nymphs, males and females of *Rhipicephalus microplus*.

Please download the files at:

<http://exon.niaid.nih.gov/transcriptome/Rhip_microplus/2015-07/Table_S1.zip>

The webpage is best accessed with the following browsers: Google Chrome, Mozilla Firefox and Internet Explorer.

Unzip the file and keep the folder “links” inside the Table_S1 directory, in order to open properly the hyperlinks from Excel spreadsheet. It is highly recommended that the Excel file be opened from a Windows-based computer (MAC or Linux users may experience some difficulty to open the hyperlinks).

Box 1 provides an explanation about the organization of the transcriptome data of RMallHxN dataset throughout the Excel spreadsheet.

**Box 1. Legends for RMallHxN dataset:** each row represents an assembled contig and each column represents a feature annotation/information about the contig. Assembled contigs may contain ESTs from different libraries. The meaning of the most columns annotation is intuitive; however some of them need additional information, which is listed below.

| **Column** | **Title** | **Description** |
| --- | --- | --- |
| M | Comments | The most significant blast hit against all databases used is placed here. The column O (Dbase) lists the database, in which lowest E-value (Column P) was obtained. |
| N | Class | Functional classification according to collection of blast results. Abbreviations of functional classes are listed in Box 2. |
| Q-X | *Rm* | *Rm* stands for the 8 cDNA libraries (transcriptome). The numbers in rows represent the amount of ESTs found in that library. This table array (Q-X) was used to perform the chi-square test, which calculates if differential ESTs counts are statistically significant (differential expression) between RmR (ticks fed on resistant host) and RmS (ticks fed on resistant host) libraries. |
|  |  |  |
| Y-AC | Many | Chi-square test for each contig using EST count data in the comparison RmS *vs* RmR libraries. |
| AD-AU | Many | Best blast-x hit to NR-LIGHT protein database (NCBI). Since NR database is very large, we reduced the search space by defining an organism parameter. The taxa included were listed in Box 3. |
| AV-BF | Many | Best blast-x hit to BEA EST database, which contains 45,512 ESTs from *Rhipicephalus microplus* (LIBEST_014697 BEA library deposited in dbEST/NCBI database) published by Guerrero et al. (2005) [1] . |
| BG-BX | Many | Best blast-x hit to bacterial proteins (NCBI). |
| BY-CP | Many | Best blast-x hit to UniProtKB/Swiss-Prot database. |
| CQ-DM | Many | Best blast-x hit to Gene Ontology (GO). |
| DN-DX | Many | Best blast-x hit to protein domain databases, such as CDD (NCBI), PFAM and SMART. |
| DZ-EK | Many | Best blast-x hit to RM-454, which is a sialotranscriptome of ticks in the same condition described here, but obtained by next-generation sequencing (454-based RNA-seq). These new sialotranscriptome yielded more than 11,800 assembled contigs of full-length sequences (unpublished data). The raw data were deposited in SRA/NCBI (BioProject PRJNA329522). |
| EL-ES | Many | Best psi-blast hit (five iterations) to TICK-L database, which is a protein database compiled of TSA (Transcriptome Shotgun Assembly Sequence Database from NCBI) records from all tick species. Sequence proteins larger than 100 amino acids were included (“L” means large). |
| ET-FA | Many | Best psi-blast hit (five iterations) to TICK-S database, which is a protein database compiled of TSA records from all tick species. Sequence proteins smaller than 100 amino acids were included (“S” means small). |
| FB-FI | Many | Best blast-x hit to TICK-TB2 database, which is a tick protein database disclosed in the Francischetti et al. (2009) review article [2]. |
| FJ-FQ | Many | Best blast-x hit to TE (transposable elements) database from sequences deposited at NCBI. |
| FR-FU | Many | Best blast-n hit to sequences of rRNA, mitochondrial and plasmids from NCBI. |

**Box 2. Functional annotation of contigs belongs to functional classes below.**

| **Functional class** | **Abbreviation** |
| --- | --- |
| Secreted | s |
| Nuclear regulation | nr |
| Transcription factor | tf |
| Transcription machinery | tm |
| Protein synthesis machinery | os |
| Protein export machinery | pe |
| Protein modification machinery | pm |
| Proteasome machinery | prot |
| Transporters/storage | tr/storage |
| Oxidant metabolism/detoxification | detox/ox |
| Metabolism, carbohydrate | met/carb |
| Metabolism, nucleotide | met/nuc |
| Metabolism, amino acid | met/aa |
| Metabolism, lipid | met/lipd |
| Metabolism, intermediate | met/int |
| Signal transduction | st |
| Extracellular matrix/cell adhesion | extmat |
| Cytoskeletal | cs |
| Transposable element | te |
| Metabolism, energy | met/energy |
| Unknown | uk |
| Unknown, conserved | uc |
| Immunity | imm |
| Viral | vir |
| Nuclear export | ne |
| Signal transduction, apoptosis | st/apoptosis |

**Box 3. Organism search set parameter for NR protein database from NCBI.**

| **Taxa in NR-LIGHT database** |
| --- |
| "[Tribolium" "[Apis m" "[Anopheles" "[Aedes " "[Culex " "[Ixodes " "[Glossina" ["Ochlerotatus " "[Tabanus" "[Chrysops " "[Amblyomma" "[Ornithod" "[Argas " "[Rhipicephalus" "[Boophilus " "[Phlebotomus" "[Lutzomyia" "[Simulium " "[Rhodnius " "[Panstrongylus " "[Triatoma " "[Dipetalogaster""[Mus m" "[Nasonia " "[Strongylocentrotus" "[Daphnia " "[Homo sa" "[Arabidopsis" "[Escherichia" "[Pseudomonas" "[Streptococcus" "[Acyrthosiphon" "[Pediculus" "[Ciona " "[Danio " "[Caenorhabditis el" "[Drosophila mela" "[Plasmodium" "[Haemaphysalis" "[Cimex " "[Rickettsia " "[Asaia " "[Klebsiella " "[Serratia " "[Enterobacter " "[Trypanosoma " "[Leishmania " "virus]" "[Saccharomyces" "[Neurospora" "[Aplysia " "[Babesia " "[Toxoplasma" "[Nocardia " "[Rhodococcus " "[Streptomyces " "[Ceratitis " "[Hyalomma " "[Brugia" "[Branchiostoma " [Bos ta" "[Gallus g" "[Hydra " "[Oriza sa" "[Nephila " "[Titus " "[Sus sc" "[Rattus ra" "[Canis fa" "[Argiope" "[Araneus " "[Acanthoscurria " "[Agelenopsis" "[Schistosoma" "[Bombyx mor" "[Bothrops " "[Ancylostoma " "[Necator " "[Bungarus " "[Crotalus " "[Hirudo " "[Desmodus " "[Xenopsylla " "[Ctenocephalides " "[Caenorhabditis elegans" |

**References**

1. Guerrero FD, Miller RJ, Rousseau M-E, Sunkara S, Quackenbush J, Lee Y, et al. BmiGI: A database of cDNAs expressed in Boophilus microplus, the tropical/southern cattle tick. Insect Biochemistry and Molecular Biology. 2005;35:585–95.

2. Francischetti IM, Sa-Nunes A, Mans BJ, Santos IM, Ribeiro JM. The role of saliva in tick feeding. Front Biosci. 2009;14:2051–88.
