# Supplementary material for: Mining a differential sialotranscriptome of Rhipicephalus microplus guides antigen discovery to formulate a vaccine that reduces tick infestations
Source: Parasit Vectors. 2017 Apr 26;10:206. doi: 10.1186/s13071-017-2136-2 (PMC5406933; doi:10.1186/s13071-017-2136-2)
Supplement: Supplementary file 3 — Antigen-specific antibody titres for Rm239 and Rm76 antigens. (DOCX 310 kb) [file 13071_2017_2136_MOESM3_ESM.docx]

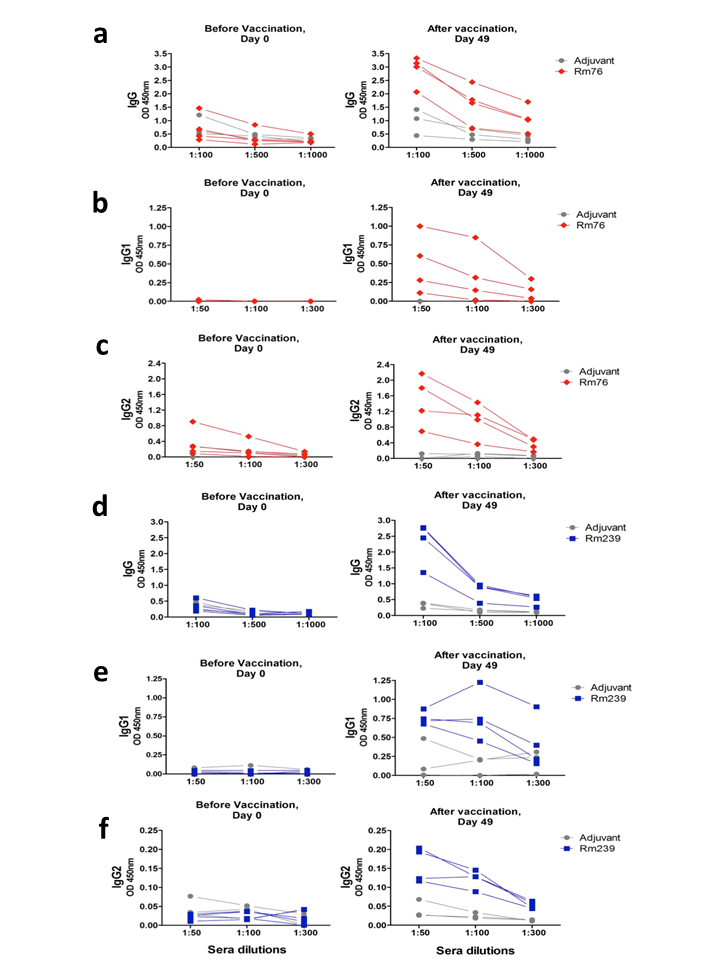


**Additional File F3: Figure S2 Antigen-specific antibody titres for Rm239 and Rm76 antigens.**

Anti-Rm76 (A, B and C) and Anti-Rm239 (D, E and F) antibody titres for total IgG (diluted to 1:100, 1:500 and 1:1000) and IgG subtypes 1 and 2 (diluted to 1:50, 1:100 and 1:300) in vaccinated calves compared to control calves injected with adjuvant alone before vaccination (day 0 of the trial) and one week after the third vaccine injection (day 49 of the trial).
